# Book Reviews

**Published:** 1915-07

**Authors:** 


### Book Reviews.

---

CANCER. By L. Duncan Bulkley, M. D., of the New York Skin and Cancer Hospital. Cloth. 230 pages. Price, $1.50. New York: Paul B. Hoeber. 1915.

This volume is made up of a series of six consecutive lectures which were delivered by the author at the New York Skin and Cancer Hospital. It is a presentation of Dr. Bulkley's personal views as to the cause and treatment of cancer. Citations are made to prove that many theories previously advocated as to the etiology of cancer are without support, and are being generally rejected. The author disagrees with the expressed views of other cancer experts, but the book is interesting and worthy of the perusal of those who are interested in this subject.

---

THE PRACTICAL MEDICINE SERIES, GENERAL MEDICINE. Volume I, Series 1915. This volume is complete and may be purchased separately by those interested in this special subject only. Price $1.50. Price of the series of ten volumes, $10.

This year-book in medicine, as the others, consists of abstracts of the most valuable articles in the principal journals for 1915. The general subjects followed are infectious diseases and diseases of the lungs, heart, arteries, blood; the ductless glands; the kidneys; and diseases of metabolism.

Plates and diagrams are used whenever necessary to explain the text. The book contains nearly four hundred pages.

---

THE PRACTICAL MEDICINE SERIES, VOL. I.—GENERAL MEDICINE. By Frank Billings, M. S., M. D., and J. H. Salisbury, A. M., M. D., Chicago. Under editorial direction of Charles L. Mix, A. M., M. D. 399 pages, illustrated. Series 1915. Published by Year-Book Publishers, 327 S. La Salle Street, Chicago, Ill. Price per set (10 volumes), $10; this volume separately, $1.50.

This is the first volume of the present year's series and includes the last year's progress in infectious diseases, such as tuberculosis, pneumonia, poliomyelitis, smallpox, rheumatism, pellagra, etc.; diseases of the lungs—bronchitis, asthma, etc.; heart—diagnosis, symptomatology, treatment, cardiac neuroses, congenital disease, angina pectoris, endocarditis, mitral stenosis, chronic myocardial disease, etc.; diseases of the arteries, blood and blood-making organs, ductless glands, dietetic errors, diabetes, gout and diseases of the kidneys.

This series is published primarily for the general practitioner; at the same time the arrangement in several volumes enables those interested in special subjects to buy only the parts they desire. This volume is embellished with quite a number of interesting and helpful illustrations.

---

Fever—Its Thermotaxis and Metabolism. By Isaac Ott, A. M., M. D., Professor of Physiology, Medico-Chirurgical College, Philadelphia; member of American Physiological Society; Consulting Neurologist Norristown Asylum, etc. New York City, 1914. Paul B. Hoeber, Publisher, 67-69 East Fifty-ninth Street. Cloth, 166 pages. Price $1.50 net.

A series of three lectures elucidating the author's long-continued researches into calorimetry and his localization of a thermogenic center in the corpus striatum. Thermolysis is taken up in the second lecture, the studies being largely based upon the author's calorimeter for man. Thermotaxis and metabolism, especially as illustrated in malarial fever, concludes a volume which should interest the physiologist and research worker. The work is not clinical, although it has indirect clinical bearings.—*The Medical Council.*

